# Multidimensional analysis of pulmonary tuberculosis epidemiological characteristics in Jining City from 2010 to 2024: an integrated study based on spatial clustering, trend regression, and age–period–cohort modeling

**DOI:** 10.3389/fpubh.2026.1727864

**Published:** 2026-03-31

**Authors:** Qinglin Li, Huimin Gu, Jinming Su, Yongrui Ling, Shuyue Yang, Jun Wang, Wei Liu, Wenguo Jiang, Mingzhen Zhang, Ling Xue, Jing Zhang, Wenjun Wang

**Affiliations:** 1 Public Health Nursing Institute, Weifang Nursing Vocational College, Weifang, China; 2 School of Public Health, North China University of Science and Technology, Tangshan, China; 3 Jining Patriotic Health and Health Promotion Center, Jining, China; 4 School of Public Health, Guangdong Medical University, Dongguan, China; 5 Department of Infectious Disease Control, Jining Center for Disease Control and Prevention, Jining, China; 6 Medical Quality Supervision Department, Jining Center for Disease Control and Prevention, Jining, China; 7 School of Public Health, Jining Medical University, Jining, China

**Keywords:** age–period–cohort model, characteristics, epidemiological, Joinpoint regression model, pulmonary, spatiotemporal clustering, tuberculosis

## Abstract

**Objective:**

To understand the spatiotemporal distribution characteristics, epidemiological features, and long-term trends in the incidence of pulmonary tuberculosis in Jining City from 2010 to 2024, and to provide scientific evidence for the formulation of targeted prevention and control strategies.

**Methods:**

Spatial autocorrelation and spatiotemporal scan analyses were used to study the spatial distribution characteristics and spatiotemporal clustering of tuberculosis incidence in Jining City. The Joinpoint regression and age–period–cohort models were employed to analyze the long-term trends of tuberculosis incidence, as well as the impact of age, period, and birth cohort.

**Results:**

From 2010 to 2024, 43,816 tuberculosis cases were reported in Jining City, with an overall average annual incidence rate of 35.31 per 100,000. Global spatial autocorrelation showed that cases were randomly distributed in most years, with significant clustering only in 2010, 2016, and 2019. Local autocorrelation analysis indicated that the central and southwestern areas of Jining City exhibited low-low clustering, while the northern and northeastern areas showed high-low and high-high clustering. Spatiotemporal scan analysis further detected various spatiotemporal clusters in certain regions, including Sishui County, Qufu City, Wen Shang County, and Jiaxiang County. The Joinpoint regression model indicated a significant downward trend in incidence across all groups, with the fastest average decrease observed among males. The age–period–cohort model showed that age, period, and cohort all significantly influenced the incidence of tuberculosis. Net drift and local drift values confirmed a declining trend, with the slowest decrease in the 20–24 age group. The age effect demonstrated an initial rise followed by a decline in incidence rate, peaking in the 20–24-year age group; the period effect showed a declining trend in tuberculosis risk over time in Jining City; the cohort effect indicated that the later the birth cohort, the lower the risk of developing tuberculosis.

**Conclusion:**

Although the incidence of tuberculosis in Jining City is on a declining trend, localized hotspots and certain spatiotemporal clusters have been identified in specific counties and periods. Furthermore, the disease incidence is influenced by factors such as age, period, and cohort. Further targeted measures should be taken to curb disease spread.

## 1 Introduction

Pulmonary tuberculosis (PTB) is a chronic infectious disease caused by *Mycobacterium tuberculosis*. It is one of the most widespread diseases in the world, second only to AIDS, and is a major cause of disease-related deaths globally (1). China not only has a high burden of tuberculosis but is also among the countries most severely affected by drug-resistant tuberculosis (2). According to the World Health Organization (WHO) “Global Tuberculosis Report 2024” China is one of the 30 countries with the highest burden of pulmonary PTB, ranking third among high-burden PTB countries (3). As a legally designated Category B infectious disease in China, PTB consistently ranks among the top Category B infectious diseases in terms of incidence and mortality rates. Although China’s PTB incidence rate has decreased in recent years, there is still a considerable gap in reaching the WHO’s goal of “ending tuberculosis” by 2035. In addition, owing to China’s large population and persistently high infection rate, the situation remains extremely severe, and PTB poses a serious threat to public health.

Jining, located in eastern China, is one of the most important cities in Shandong Province. This city has a large population of over 8 million permanent residents, convenient transportation, and abundant tourism resources. Located at the “golden coordinate” marking the north–south divide of the Beijing-Hangzhou Grand Canal, it experiences high population movement and bustling commercial activity. Simultaneously, Jining is also a well-known coal industry base in China, with annual coal production consistently reaching tens of millions or even hundreds of millions of tons, holding a significant place in the national energy landscape (4). High population density, large-scale mobility, and occupational health risks associated with the coal industry collectively increase the risk of PTB in this area, presenting major challenges to disease prevention and control.

Although there are many similar studies at the national level, a systematic analysis of the long-term and multi-dimensional epidemiological patterns of Jining—a region characterized by unique population movement and industrial features—remains lacking. To further investigate the spatiotemporal characteristics, incidence trends, and features of PTB in Jining, this study employed spatial autocorrelation and spatiotemporal scan analyses to explore the spatiotemporal distribution of PTB incidence in Jining from 2010 to 2024. Geographic Information Systems (GIS) and spatiotemporal cluster analyses are widely used in public health, especially in infectious disease research. These methods help identify disease clusters and dynamically visualize incidence rates and clustering trends over time and space (5). In addition, this study used the Joinpoint regression and Age–Period–Cohort (APC) models to systematically analyze the temporal trends of PTB incidence in the region and to assess the impact of age, period, and birth cohort on population risk, thereby providing scientific evidence for developing targeted PTB prevention and control strategies in Jining.

## 2 Data and methods

### 2.1 Data sources and study area

**Figure 1:**
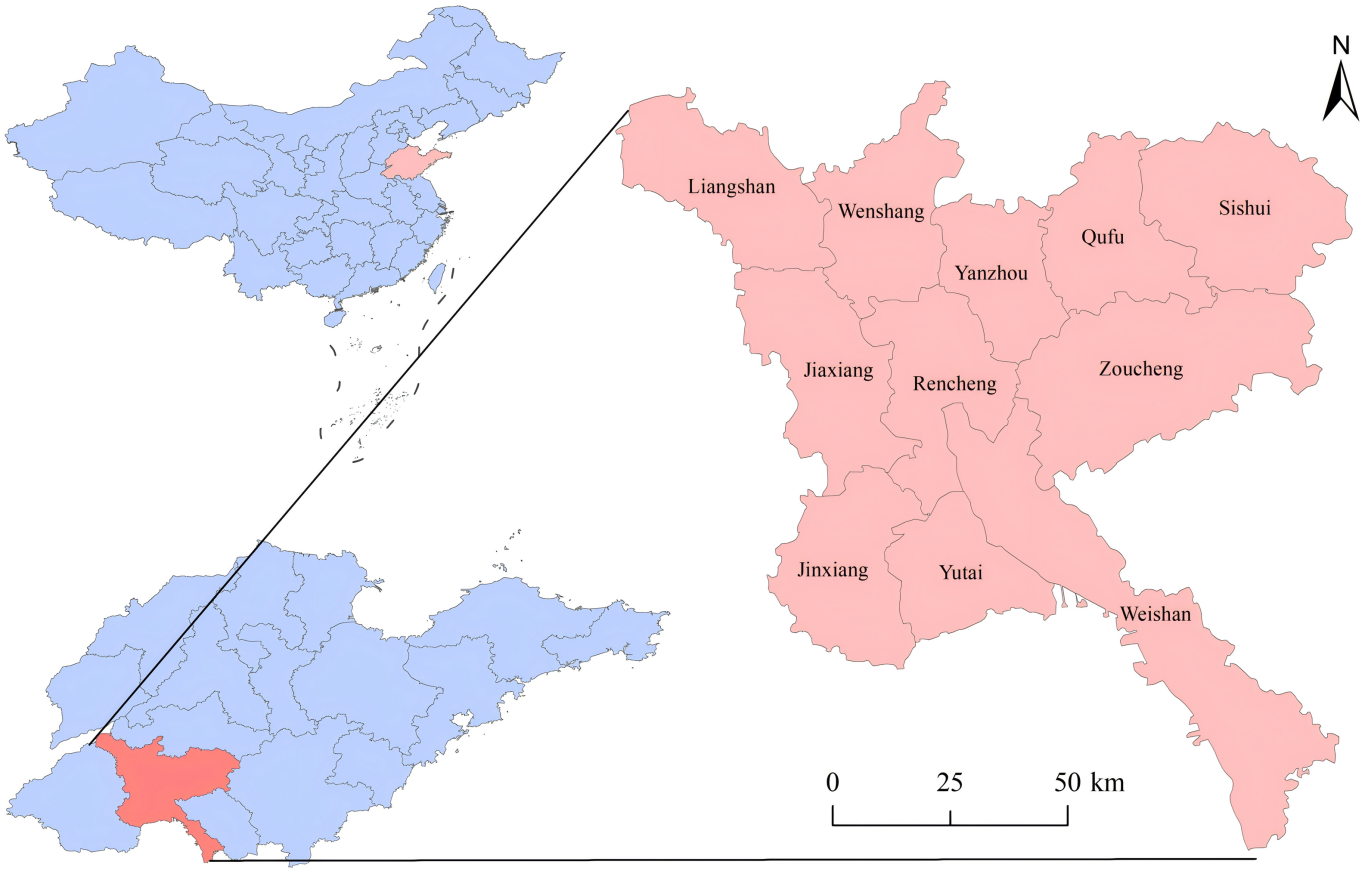
Geographical location of Jining City.

This study obtained data on PTB incidence in Jining from 2010 to 2024 through the National Infectious Disease Reporting Information Management System (NIDRIMS), established in 2004 and covering all medical institutions in China. Demographic information was obtained from the basic information system module within NIDRIMS and the “Jining Statistical Yearbook (2010–2024)”, published by the Jining Bureau of Statistics. Jining City is composed of 11 counties (urban districts): Liangshan, Jiaxiang, and Jinxiang counties in the western region; Wenshang, Rencheng, Yutai, and Weishan counties in the central region; and Yanzhou, Qufu, Zoucheng, and Sishui counties in the eastern region. The geographical location and regional divisions of Jining in China are shown in Figure 1. This study selected “county (urban district)” as the research unit of analysis.

### 2.2 Research methods

#### 2.2.1 Spatial autocorrelation analysis

Spatial autocorrelation analysis consists of global and local spatial autocorrelation analyses. The global autocorrelation analysis uses the global Moran’s I index to test the spatial clustering of variables in the entire study area, with a range of [−1, 1], where *I* > 0 indicates a positive spatial correlation, *I* < 0 indicates a negative correlation, and *I* = 0 indicates a random spatial distribution (6). Regardless of the presence of global spatial autocorrelation, local spatial autocorrelation analysis can be used to identify potential local clustering patterns. Local spatial autocorrelation analysis (local indicators of spatial association, LISA) visualizes the aggregation between a locality and its neighboring areas by creating LISA maps that show four clustering models: high-high, high-low, low-high, and low-low. High-high indicates that PTB incidence is high both in the area and its neighbors; high-low means the area has high incidence while its neighbors are low; low-high and low-low are opposite to the former, respectively (7). GeoDa 1.22 software was used for both global and local spatial autocorrelation analyses.

#### 2.2.2 Spatiotemporal scan analysis

Spatial scan statistics are tools for detecting disease clusters in space and time. Spatiotemporal scanning not only reveals the changing trends of spatial clusters over time but also determines the relative risk of the cluster areas, providing a more accurate location of spatial clusters (8). In this study, SaTScan 10.2.5 software was used, with counties (districts) as spatial units and months as time units, to construct a Poisson distribution model for scanning. The logarithmic likelihood ratio (LLR) statistic was used to describe the degree of abnormal clustering within a window by comparing the observed and expected values, and the classification of clusters was judged based on the LLR and relative risk (RR) values (5). The LLR is calculated as follows:


$$\mathrm{LLR}=\log{\left(c/n\right)}^{c}{\left[(C-c)/(C-c)\right]}^{\left(c-c\right)}$$

where C is the total number of cases, and c and n are the observed and expected cases within the window, respectively. Monte Carlo simulation (*α* = 0.05, with 999 replications) was used to estimate the *p*-value (9).

#### 2.2.3 Joinpoint regression analysis

Joinpoint regression analysis, using segmented linear modeling and permutation tests, overcomes the limitations of traditional linear regression in fitting complex nonlinear patterns and can objectively identify inflection points in time series data. It has been widely applied in epidemiological research (10). This study used Joinpoint 5.2.0 software to analyze the trend in standardized PTB incidence rates in Jining from 2010 to 2024 (standardized against the 2020 national census population) and calculated the average annual percentage change (AAPC) with 95% confidence intervals (CI). If the optimal model is a segmented function, the annual percentage change (APC) for each segment is calculated. APC > 0 indicates an increasing trend, APC < 0 a decreasing trend, and APC = AAPC indicates no inflection in the incidence rate; the significance level was *α* = 0.05 (11).

#### 2.2.4 APC model analysis

The APC model is a statistical model used to analyze the interactive influence of age, period, and cohort, quantifying the independent effect of each dimension on disease incidence, and analyzing disease trends (12). This study utilized an online tool provided by the U.S. National Cancer Institute^1^ to build the model. Due to limitations in demographic data and to ensure a sufficient number of cases in each age-period stratum, the study grouped age, period, and cohort by 5-year intervals, resulting in 18 age groups (<5 years, 5–9 years, ≥85 years), 3 periods (2010–2014, 2015–2019, 2020–2024), and 20 birth cohorts (1925–1929, 1930–1934…2020–2024). The middle period and cohort within each interval were chosen as the reference groups. Parameter tests were performed using the Wald *χ*^2^ test at a significance level of *α* = 0.05 (13).

The core output indicators of the APC model include: Net Drift, which is the overall annual percentage change in incidence over time, adjusted for period and cohort effects; Local Drift, which is the annual percentage change in incidence by age group, adjusted for period and cohort effects; Longitudinal Age Curve, which is the incidence rate in specific age groups adjusted for period deviation; Period Rate Ratio (PRR), which is the incidence rate in each period relative to the reference period, adjusted for age and cohort effects; and Cohort Rate Ratio (CRR), which is the incidence rate in each birth cohort relative to the reference cohort, adjusted for age and period effects (14, 15).

## 3 Results

### 3.1 Epidemic status

**Figure 2:**
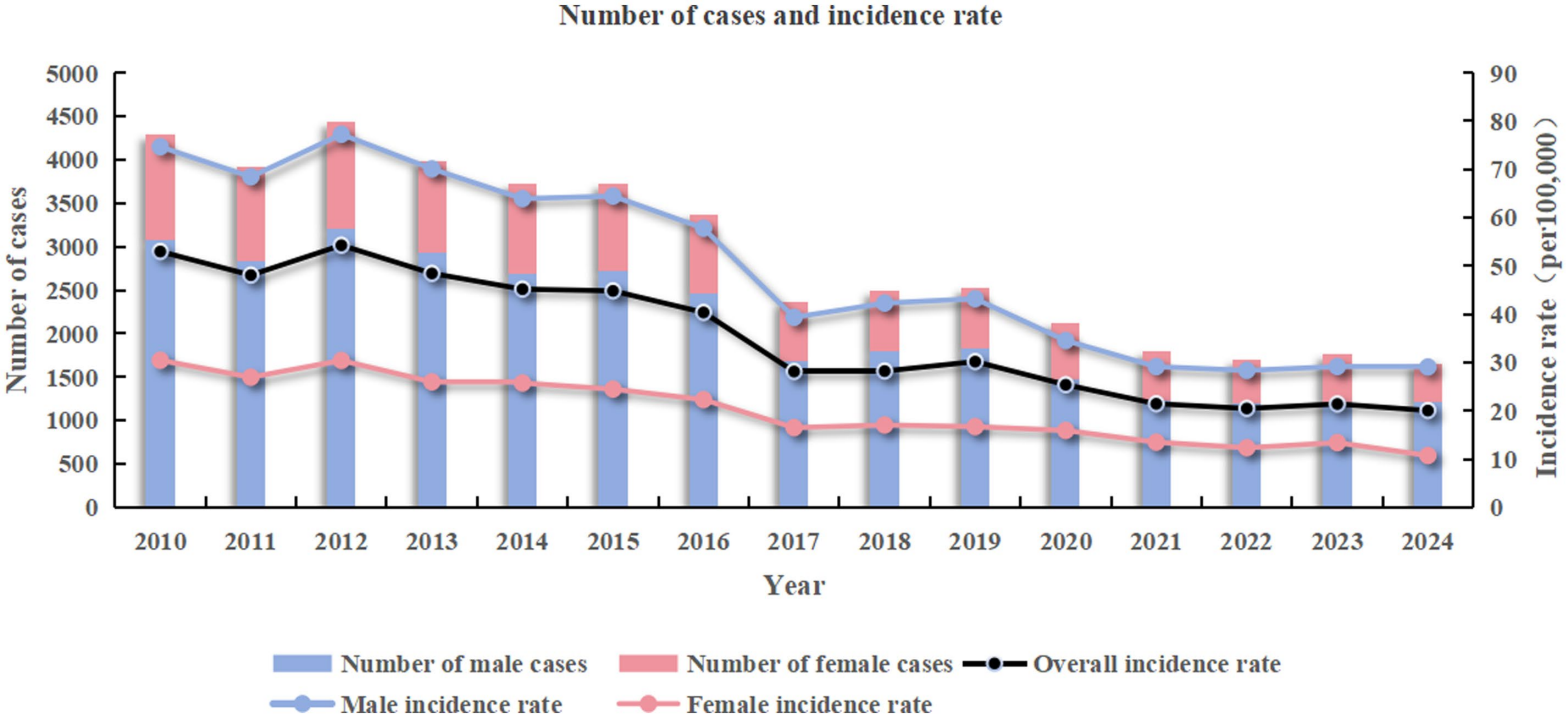
Number and incidence rate of PTB in the whole population and by sex in Jining from 2010 to 2024.

From 2010 to 2024, a total of 43,816 cases of PTB were reported in Jining, with an overall average annual incidence rate of 35.31 per 100,000, and an age-standardized incidence rate of 35.69 per 100,000. The average annual incidence rate among males was 50.12 per 100,000, with an age-standardized incidence rate of 50.14 per 100,000, while for females it was 20.18 per 100,000, with an age-standardized incidence rate of 19.14 per 100,000. Among these, there were 31,553 male cases and 12,263 female cases, giving a male-to-female ratio of 2.57:1 (as shown in Figure 2).

### 3.2 Spatial distribution characteristics

**Figure 3:**
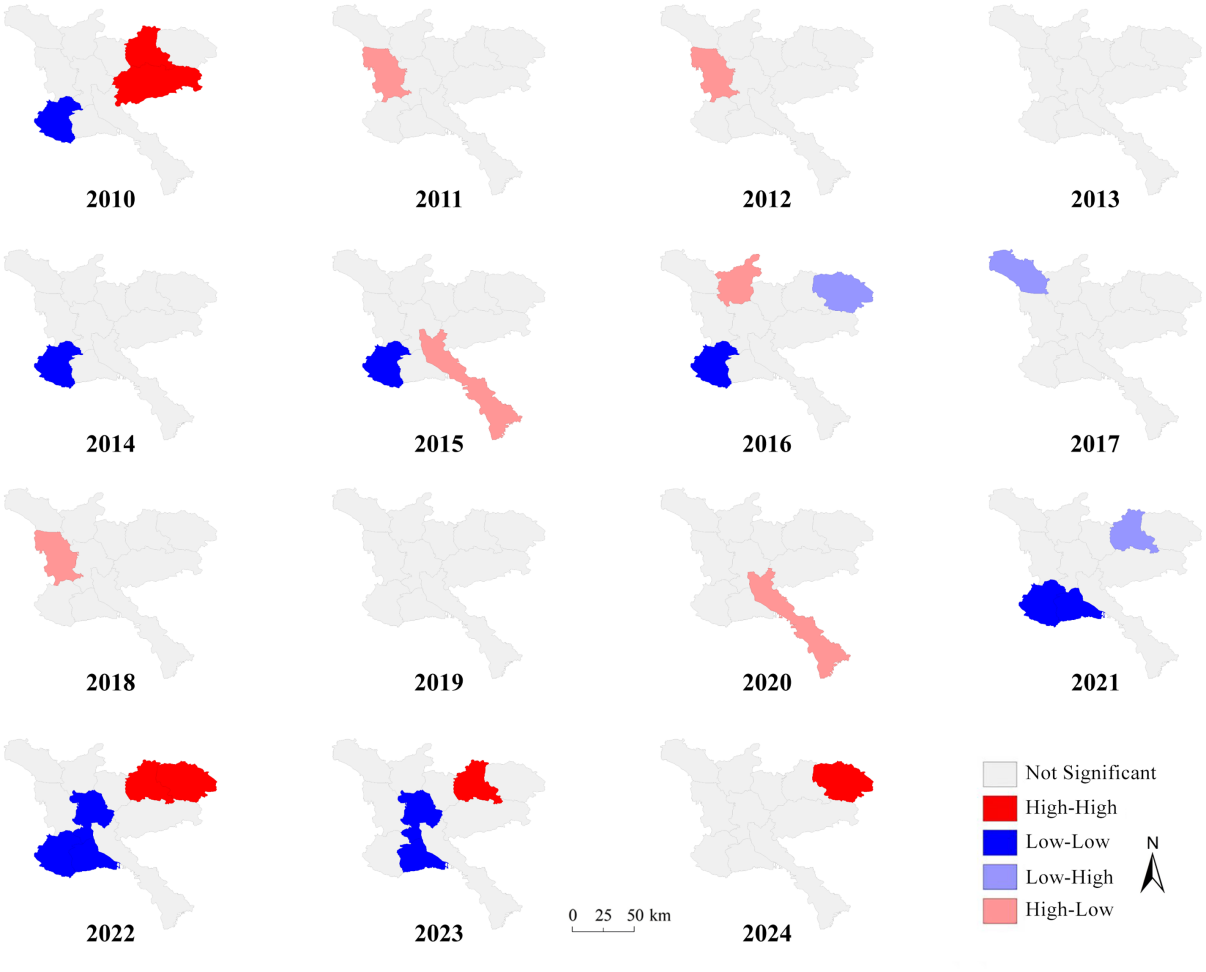
Local autocorrelation results of PTB in Jining from 2010 to 2024.

From 2010 to 2024, global Moran’s I was statistically significant only in 2010, 2016, and 2019, indicating a clustered distribution in 2010 and a dispersed distribution in 2016 and 2019. In the remaining years, there was no statistical significance, suggesting that there was no spatial correlation in PTB incidence, indicating that cases were randomly distributed overall (see Table 1). Further local spatial autocorrelation analysis revealed, according to the LISA cluster map (Figure 3), that except for 2011 and 2013, there were localized cluster areas in other years. Overall, the central and southwestern regions of Jining (including Jiaxiang County, Rencheng District, Jinxiang County, and Yutai County) showed a low-low pattern, whereas the northern and northeastern regions (including Wenshang County, Qufu City, and Sishui County) mainly exhibited high-low and high-high clustering.

**Table 1:** Global autocorrelation results of PTB in Jining from 2010 to 2024.

Year	Moran’s I	*Z*-value	*p*-value
2010	0.308	2.166	0.023
2011	0.051	0.798	0.213
2012	−0.217	−0.689	0.264
2013	−0.183	−0.456	0.327
2014	−0.274	−0.988	0.166
2015	−0.160	−0.293	0.410
2016	−0.393	−1.5844	0.039
2017	−0.161	−0.328	0.405
2018	−0.296	−1.044	0.148
2019	−0.415	−1.579	0.021
2020	−0.337	−1.277	0.070
2021	−0.185	−0.436	0.364
2022	0.101	1.127	0.126
2023	−0.178	−0.453	0.359
2024	−0.211	−0.636	0.279

### 3.3 Spatiotemporal scan analysis

A spatiotemporal scan of PTB cases reported in each county (city, district) of Jining from 2010 to 2024 showed evidence of spatiotemporal clustering. Eight clusters were identified: two type I, three type II, and three type III clusters. Except for Yanzhou District and Zoucheng City, all other areas in Jining City showed varying degrees of clustering, with the main clustering areas being Sishui County, Qufu City, Wenshang County, and Jiaxiang County over the period from 2010 to 2020 (see Table 2 for details).

**Table 2:** Spatiotemporal scanning results of PTB in Jining from 2010 to 2024.

Clusters type	Gathering time	Gathering area	Observed cases	Expected cases	RR	LLR	*p*
I	2010/1–2013/9	Shishui, Qufu, Jiaxiang	5,045	3,065	1.73	583.11	<0.001
I	2013/1–2016/6	Liangshan, Wenshang, Jiaxiang	4,174	2,746	1.57	344.93	<0.001
II	2010/1–2012/9	Liangshan, Wenshang, Jiaxiang	3,203	2,267	1.45	181.42	<0.001
II	2013/11–2017/5	Weishan	1,382	815	1.72	166.51	<0.001
II	2013/11–2016/4	Shishui, Qufu, Jiaxiang	2,894	2054	1.44	160.63	<0.001
III	2010/1–2013/1	Yutau, Jinxiang, Rencheng	3,012	2,350	1.3	90.74	<0.001
III	2018/1–2020/12	Shishui	798	580	1.38	36.97	<0.001
III	2018/1–2020/8	Jiaxiang	859	656	1.31	28.78	<0.001

### 3.4 Joinpoint regression analysis of incidence trends

**Figure 4:**
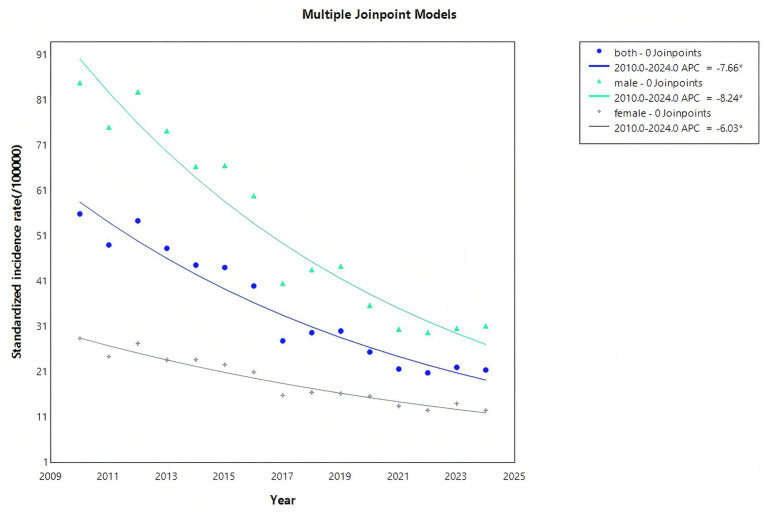
Joinpoint regression results of PTB in Jining from 2010 to 2024.

The Joinpoint regression model did not identify significant inflection points in the incidence trends for the overall population, males, or females, indicating a monotonic trend in the incidence rates from 2010 to 2024 for all groups (see Figure 3). The overall annual percent change APC = AAPC was −7.66% (95% CI: −8.81% to −6.50%), with the APC for males at −8.24% (95% CI: −9.50% to −6.96%) and for females at −6.03% (95% CI: −6.97% to −5.08%). This demonstrates a significant downward trend in incidence rates for all groups, with the fastest decline rate among males (Detailed results can be found in the Supplementary Table S2) (Figure 4).

### 3.5 APC analysis of incidence rate trends

#### 3.5.1 Net drift and local drift

**Figure 5:**
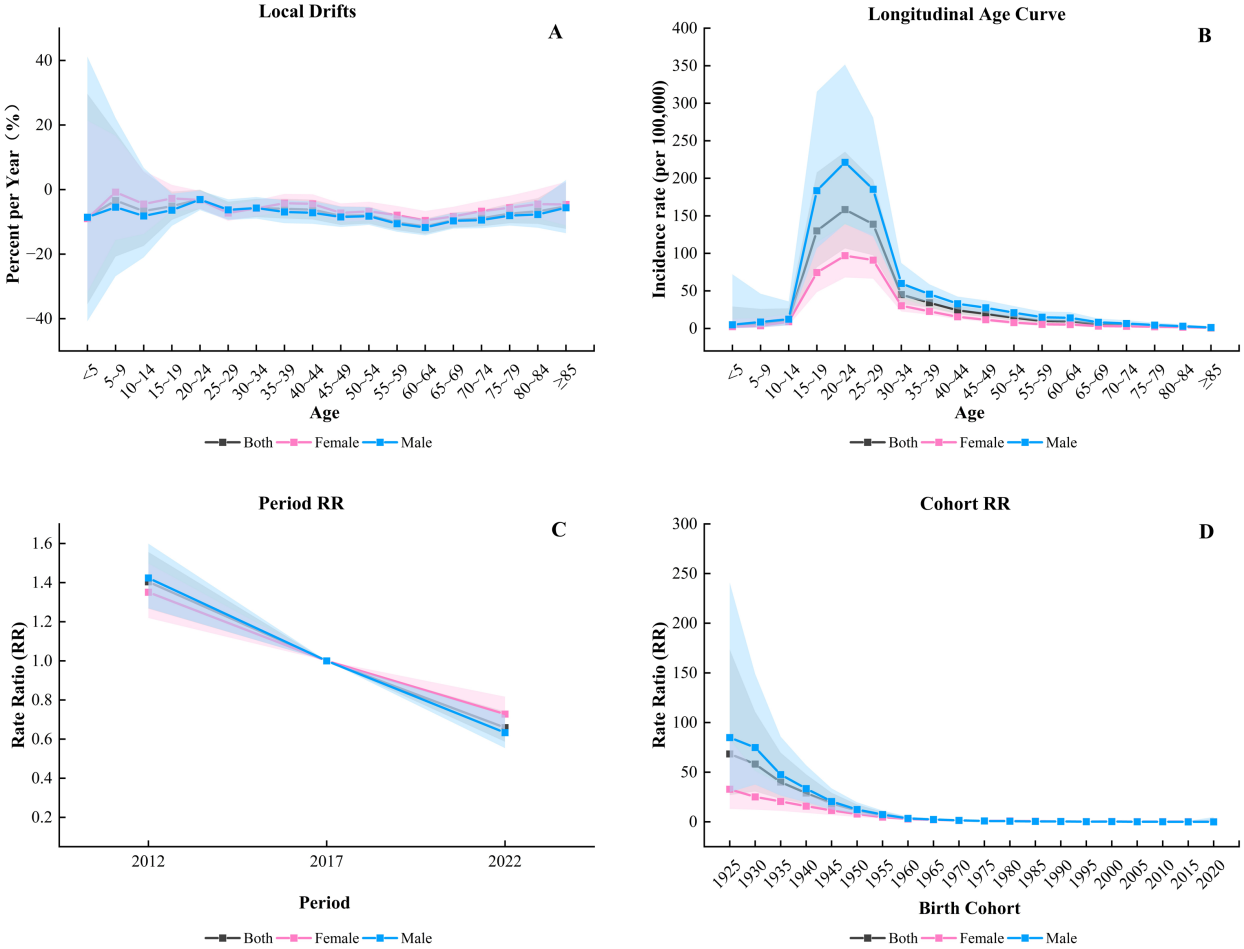
APC model results of PTB in Jining from 2010 to 2024. **(A)** Local drifts; **(B)** age effect; **(C)** period effect; **(D)** cohort effect.

The net drift for PTB incidence in the overall population of Jining was −7.28% (95% CI: −8.56% to −5.98%), with males at −7.79% (95% CI: −9.31% to −6.25%) and females at −5.99% (95% CI: −7.19% to −4.79%). Among local drifts, only the overall (*χ^2^* = 32.99, *p* = 0.016) and male (*χ^2^* = 29.15, *p* = 0.048) drifts were statistically Generally, the changes in local drifts for the entire population and for males were similar, with the slowest rate of decrease in the 20–24 age group overall: −3.04% (95% CI: −5.94% to −0.05%);males: −3.08% (95% CI: −6.36% to −0.32%). Subsequently, the incidence rates showed a fluctuating downward trend with increasing age, reaching the fastest decrease in the 60–64 age group overall: −11.38% (95% CI: −13.72% to −8.98%); males: −11.75% (95% CI: −14.17% to −9.26%), and then trended upward again (see Figure 5A).

#### 3.5.2 Age effect

After controlling for period and cohort effects, the longitudinal age curve indicated that the incidence rate of PTB in the overall population, males, and females first increased and then decreased, peaking in the 20–24 age group. The incidence rate among males was higher than that of the overall population and females (see Figure 5B), at 158.42/100,000 (95% CI: 106.62–235.39) for overall, 221.30/100,000 (95% CI: 139.32–351.54) for males, and 96.96/100,000 (95% CI: 67.89–138.48) for females. Subsequently, the incidence rate declined with increasing age.

#### 3.5.3 Period effect

Taking the period effect from 2015 to 2019 as the reference (RR = 1), and after adjusting for age and cohort effects, the results showed that the PTB risk for the overall population, males, and females all declined over time (see Figure 5C). The period relative risks (PRR) for 2010–2014 were 1.40 (95% CI: 1.27–1.56) for the overall population, 1.42 (95% CI: 1.27–1.60) for males, and 1.35 (95% CI: 1.22–1.50) for females. For 2020–2024, the PRRs were 0.66 (95% CI: 0.59–0.74) for the overall population, 0.63 (95% CI: 0.55–0.72) for males, and 0.73 (95% CI: 0.65–0.82) for females.

#### 3.5.4 Cohort effect

Using the 1975–1979 birth cohort as the reference group (RR = 1) and adjusting for age and period effects, the results showed RR values greater than 1 for the 1925–1974 cohorts and lower than 1 for the 1979–2024 cohorts. The later the birth year, the lower was the risk of developing PTB (see Figure 5D). Those born in 1925–1929 had the highest cohort relative risk (CRR), at 68.43 (95% CI: 26.98–173.56) for the overall population, 84.92 (95% CI: 29.95–240.77) for males, and 32.92 (95% CI: 13.01–83.27) for females. Subsequently, the risk decreased, reaching its lowest point in the 2020–2024 cohort, with CRRs of 0.07 (95% CI: 0.002–2.60) overall, 0.05 (95% CI: 0.001–4.86) for males, and 0.09 (95% CI: 0.004–1.92) for females. Before 1960, the risk declined rapidly with later birth years, and after that, the decline slowed. By sex, males had the highest risk in all birth cohorts, but their risk also declined the fastest (All complete data of the APC model can be found in Supplementary Table S1).

## 4 Discussion

This study systematically and comprehensively revealed the spatial, temporal, and demographic distribution characteristics, as well as the epidemic trends and patterns of PTB incidence in Jining from 2010 to 2024, by analyzing the features, trends, and age–period–cohort effects of PTB cases in the region. In terms of temporal distribution, Joinpoint regression analysis showed that while the PTB incidence rate in Jining slightly fluctuated over the past 15 years, it generally declined. The trends among males and females mirror the pattern observed in the general population, with the average decline rate in males being slightly faster than that in females and the overall population. This trend is further confirmed by the net drift value and period effect from the APC model; specifically, the net drift values for the total population, males, and females in Jining ranged from −5.99% to −7.79%, indicating an overall annual decrease in incidence over time. The period effect also demonstrated that when using the mid-term period as a reference, the risk of PTB incidence in Jining steadily declined as the years progressed. Nowadays, this continuous decrease in PTB incidence is closely related to the national emphasis on PTB prevention and control: China has successively introduced the *National Tuberculosis Control Plans* in 1997, 2001, 2016, and 2024, incorporated PTB into the New Rural Cooperative Critical Illness Insurance in 2012, and in 2019, the National Healthcare Security Administration set anti-tuberculosis outpatient drug costs to “zero out-of-pocket.” These policies and official documents have significantly alleviated the financial burden on patients with PTB and ensured timely diagnosis and treatment, playing a crucial role in steadily reducing PTB incidence and risk (16–18). Furthermore, ongoing improvements in living conditions, continuous advancements in medical care, and growing public awareness of health have together established a comprehensive disease prevention and control system, further disrupting PTB transmission chains and enabling a transition from “treatable” to increasingly “preventable and controllable” tuberculosis (19).

From the perspective of population distribution, the cohort effect results of the APC model showed that individuals born earlier had a higher risk of developing PTB, whereas those born later had a lower risk. This phenomenon arises from environmental and social changes, as different birth cohorts are exposed to different risk factors. Before 1960, China experienced war, social upheaval, and an incomplete societal system; people born during this period faced a lack of medical resources and poor nutrition in childhood, resulting in a higher cumulative risk of infection and disease. Subsequently, with improvements in living standards, wider coverage of BCG vaccination, and advancements in diagnostic technology, the lifetime risk of developing PTB has continuously declined (20). This diminishing cohort effect reveals the long-term suppressive impact of socioeconomic development and public health interventions on the disease burden. Though the number of cases in the earliest and latest birth cohorts was relatively small and the risk estimates for marginal cohorts may be somewhat unstable, the overall observed trend still provides important population anchors for future targeted prevention and control strategies. Furthermore, the age effect results indicate that for the overall population, both males and females have the highest incidence in the 20–24 age group. Similarly, the local drift value also peaked in the 20–24 age group (To provide a clearer view of the local offset values across different age groups, we included an image in the Supplementary material that removes the confidence intervals and retains only statistically significant values, See Supplementary Figure S1 for details), indicating that the rate of decline in incidence was the slowest in this age range. This is similar to the findings of Sarawati et al. (21) on the national PTB epidemic, where the 20–25 age group showed a moderate peak in incidence nationwide. This may be due to the fact that this age group mainly includes students or new employees, and schools and workplaces often screen for PTB during student enrollment or employee health checks, at which time latent cases are identified. However, while nationwide, the peak in PTB incidence in this age group is more concentrated among women, in Jining, the incidence rate among men in this age range is significantly higher than that among women. Additionally, the decline in incidence rate was slowest in this age group, suggesting the possible existence of a relatively stable, persistent risk exposure. Jining City is one of China’s major coal-producing bases, with a long history of mining and a large workforce of young male miners. The tuberculosis hotspots identified in this study spatially overlapped with the main coal-producing areas (such as Jiaxiang and Weishan). The occupational environment of the coal mining industry may be an important contributor to the unique age-sex patterns of tuberculosis in Jining City (22–25).

From the perspective of regional distribution, the results of the LISA map and spatiotemporal scan analysis were similar. To ensure greater rigor, we additionally used FleXScan software to conduct a flexible shape scan analysis based on administrative boundaries in order to overcome the limitation of spatio-temporal scans, which can only use cylindrical scanning (The results are shown in Supplementary Table S3). We found that the results of all three methods were largely consistent: tuberculosis cases in Jining City were mainly concentrated in areas near Wenshang County, Jiaxiang County, Sishui County, and Qufu City. In particular, Sishui and Qufu have shown high-high clustering in recent years, and these are also classified as primary and secondary clustering areas. We hypothesized that the high incidence of Qufu may be related to substantial population mobility. Qufu is known as the “Jerusalem of the East” and is the birthplace of Confucius, a world-renowned philosopher. As a famous tourist city and a World Cultural Heritage site, Qufu has attracted a large number of domestic and international tourists in the recent years (26). The dense flow of tourists increases the risk of PTB transmission, and both areas have considerable labor migration and population mobility, which may facilitate the introduction of pathogens and local transmission (27). Ecological analyses conducted by Chinese scholars across 31 provinces have confirmed that factors, such as economic conditions and medical resources, are closely linked to tuberculosis incidence (16). From a socioeconomic perspective, certain regions of Sishui exhibit relatively lagging economic development and weak medical resources, which may lead to insufficient local health awareness and inadequate dissemination of knowledge on prevention and control. This situation may result in delayed medical treatment and irregular care among patients, collectively increasing the incidence of PTB in these areas.

Our study still has several limitations. First, the analysis did not account for undiagnosed, unreported, or non-case definition-compliant cases, which may be more pronounced in regions with relatively weaker medical resources, lower health awareness, or limited diagnostic capacity, likely resulting in poorer reporting integrity and affecting the precision of our spatial cluster results and incidence trends to some extent. Second, APC and spatial analyses were conducted at the aggregated group level. Although these models can identify high-risk populations and spatial clusters that are valuable for public health planning, they cannot be directly interpreted as individual-level causal relationships. For example, a high incidence among 20–24 age group men does not necessarily mean that each individual in this group contracted the disease due to occupational exposure in mining. In the future, the precise causal relationship between the two requires confirmation through individual-level studies. Finally, the estimates of the APC model, especially for the oldest and youngest birth cohorts, may be unstable because of the small number of cases in these extreme groups. The wide confidence intervals observed in these groups indicate that these estimates should be interpreted as indicative of direction and magnitude rather than as precise values. Future studies with longer observation periods or larger populations are needed to obtain more stable estimates for these extreme cohorts.

To address these limitations, much remains to be explored in future research. For instance, to assess the disease burden more accurately and correct for surveillance data bias, future research should integrate individual-level survey data and strengthen active case findings in high-risk cluster areas. Additionally, to mitigate ecological fallacy and better elucidate disease etiology, follow-up research could collect individual-level occupational histories, behavioral data, and environmental data at district and county levels. Controlling for individual confounders would allow for a more accurate estimation of the independent effects of regional environmental and occupational characteristics. These efforts will collectively advance our understanding of the epidemiological features of PTB in Jining, from broad associations to more detailed mechanistic insights.

## 5 Conclusion

In summary, from 2010 to 2024, the overall trend in Jining is a declining rate and risk of PTB incidence, with high-risk cases primarily concentrated among males aged roughly 20–24, and those born earlier still facing higher lifetime risk. Incidence hotspots were mainly found in the northern and northeastern counties of Jining, including Wenshang, Qufu, Sishui, and Jiaxiang. To further curb the PTB epidemic, future prevention and control strategies should focus on these identified high-risk populations and hotspots. For populations in coal-producing areas and their young male workers, active screening and occupational health protection should be strengthened. For areas with relatively underdeveloped socioeconomic conditions and healthcare resources, governments need to optimize the allocation of resources and enhance the capacity of primary healthcare services. Ultimately, these efforts will provide a solid foundation for the full implementation of China’s Healthy China strategy.

## Data Availability

The original contributions presented in the study are included in the article/Supplementary material, further inquiries can be directed to the corresponding authors.

## Footnotes

1
https://analysistools.cancer.gov/apc
