# Supplementary material for: Multidimensional analysis of pulmonary tuberculosis epidemiological characteristics in Jining City from 2010 to 2024: an integrated study based on spatial clustering, trend regression, and age–period–cohort modeling
Source: Front Public Health. 2026 Mar 31;14:1727864. doi: 10.3389/fpubh.2026.1727864 (PMC13076588; doi:10.3389/fpubh.2026.1727864)
Supplement: Supplementary file 1 [file Supplementary_file_1.DOCX]

**Supplemental Materials**

**Table S1 Analysis results of Age-Period-Cohort Model**

| **sex_name** | **method_name** | **x_name** | **y_name** | **x_value** | **y_value** | **y_lower** | **y_upper** |
| --- | --- | --- | --- | --- | --- | --- | --- |
| Both | Net Drift | NA | NA | NA | -7.280 | -8.560 | -5.983 |
| Both | Local Drifts with Net Drift | Age | Percent per Year | <5 | -8.556 | -35.508 | 29.66 |
| Both | Local Drifts with Net Drift | Age | Percent per Year | 5~9 | -3.372 | -20.743 | 17.805 |
| Both | Local Drifts with Net Drift | Age | Percent per Year | 10~14 | -6.680 | -17.440 | 5.482 |
| Both | Local Drifts with Net Drift | Age | Percent per Year | 15~19 | -5.121 | -9.392 | -0.649 |
| Both | Local Drifts with Net Drift | Age | Percent per Year | 20~24 | -3.037 | -5.939 | -0.046 |
| Both | Local Drifts with Net Drift | Age | Percent per Year | 25~29 | -6.563 | -9.258 | -3.788 |
| Both | Local Drifts with Net Drift | Age | Percent per Year | 30~34 | -5.726 | -8.497 | -2.872 |
| Both | Local Drifts with Net Drift | Age | Percent per Year | 35~39 | -5.955 | -8.969 | -2.842 |
| Both | Local Drifts with Net Drift | Age | Percent per Year | 40~44 | -6.221 | -9.251 | -3.090 |
| Both | Local Drifts with Net Drift | Age | Percent per Year | 45~49 | -8.130 | -10.941 | -5.231 |
| Both | Local Drifts with Net Drift | Age | Percent per Year | 50~54 | -7.968 | -10.535 | -5.327 |
| Both | Local Drifts with Net Drift | Age | Percent per Year | 55~59 | -10.200 | -12.566 | -7.771 |
| Both | Local Drifts with Net Drift | Age | Percent per Year | 60~64 | -11.383 | -13.721 | -8.983 |
| Both | Local Drifts with Net Drift | Age | Percent per Year | 65~69 | -9.466 | -11.776 | -7.096 |
| Both | Local Drifts with Net Drift | Age | Percent per Year | 70~74 | -8.894 | -11.365 | -6.354 |
| Both | Local Drifts with Net Drift | Age | Percent per Year | 75~79 | -7.426 | -10.404 | -4.349 |
| Both | Local Drifts with Net Drift | Age | Percent per Year | 80~84 | -6.714 | -10.613 | -2.644 |
| Both | Local Drifts with Net Drift | Age | Percent per Year | ≥85 | -5.218 | -12.158 | 2.270 |
| Both | Longitudinal Age Curve | Age | Rate | <5 | 3.622 | 0.449 | 29.230 |
| Both | Longitudinal Age Curve | Age | Rate | 5~9 | 6.054 | 1.412 | 25.959 |
| Both | Longitudinal Age Curve | Age | Rate | 10~14 | 11.098 | 4.607 | 26.730 |
| Both | Longitudinal Age Curve | Age | Rate | 15~19 | 130.040 | 81.379 | 207.797 |
| Both | Longitudinal Age Curve | Age | Rate | 20~24 | 158.423 | 106.621 | 235.392 |
| Both | Longitudinal Age Curve | Age | Rate | 25~29 | 138.920 | 97.475 | 197.988 |
| Both | Longitudinal Age Curve | Age | Rate | 30~34 | 45.170 | 32.776 | 62.252 |
| Both | Longitudinal Age Curve | Age | Rate | 35~39 | 34.292 | 27.493 | 42.773 |
| Both | Longitudinal Age Curve | Age | Rate | 40~44 | 24.172 | 19.227 | 30.391 |
| Both | Longitudinal Age Curve | Age | Rate | 45~49 | 19.463 | 14.882 | 25.453 |
| Both | Longitudinal Age Curve | Age | Rate | 50~54 | 14.329 | 10.390 | 19.761 |
| Both | Longitudinal Age Curve | Age | Rate | 55~59 | 10.082 | 6.993 | 14.537 |
| Both | Longitudinal Age Curve | Age | Rate | 60~64 | 9.494 | 6.330 | 14.239 |
| Both | Longitudinal Age Curve | Age | Rate | 65~69 | 5.489 | 3.556 | 8.472 |
| Both | Longitudinal Age Curve | Age | Rate | 70~74 | 4.489 | 2.812 | 7.165 |
| Both | Longitudinal Age Curve | Age | Rate | 75~79 | 3.040 | 1.824 | 5.068 |
| Both | Longitudinal Age Curve | Age | Rate | 80~84 | 1.999 | 1.122 | 3.560 |
| Both | Longitudinal Age Curve | Age | Rate | ≥85 | 0.827 | 0.410 | 1.666 |
| Both | Period RR | Period | Rate Ratio | 2012 | 1.404 | 1.267 | 1.556 |
| Both | Period RR | Period | Rate Ratio | 2017 | 1 | 1 | 1 |
| Both | Period RR | Period | Rate Ratio | 2022 | 0.659 | 0.587 | 0.741 |
| Both | Cohort RR | Cohort | Rate Ratio | 1925 | 68.433 | 26.982 | 173.564 |
| Both | Cohort RR | Cohort | Rate Ratio | 1930 | 58.192 | 30.561 | 110.806 |
| Both | Cohort RR | Cohort | Rate Ratio | 1935 | 40.041 | 23.028 | 69.624 |
| Both | Cohort RR | Cohort | Rate Ratio | 1940 | 29.043 | 17.652 | 47.783 |
| Both | Cohort RR | Cohort | Rate Ratio | 1945 | 18.510 | 11.677 | 29.342 |
| Both | Cohort RR | Cohort | Rate Ratio | 1950 | 11.442 | 7.459 | 17.552 |
| Both | Cohort RR | Cohort | Rate Ratio | 1955 | 6.847 | 4.620 | 10.147 |
| Both | Cohort RR | Cohort | Rate Ratio | 1960 | 3.417 | 2.384 | 4.898 |
| Both | Cohort RR | Cohort | Rate Ratio | 1965 | 2.335 | 1.711 | 3.186 |
| Both | Cohort RR | Cohort | Rate Ratio | 1970 | 1.490 | 1.143 | 1.942 |
| Both | Cohort RR | Cohort | Rate Ratio | 1975 | 1 | 1 | 1 |
| Both | Cohort RR | Cohort | Rate Ratio | 1980 | 0.784 | 0.595 | 1.033 |
| Both | Cohort RR | Cohort | Rate Ratio | 1985 | 0.541 | 0.391 | 0.750 |
| Both | Cohort RR | Cohort | Rate Ratio | 1990 | 0.434 | 0.301 | 0.628 |
| Both | Cohort RR | Cohort | Rate Ratio | 1995 | 0.275 | 0.180 | 0.419 |
| Both | Cohort RR | Cohort | Rate Ratio | 2000 | 0.319 | 0.199 | 0.513 |
| Both | Cohort RR | Cohort | Rate Ratio | 2005 | 0.162 | 0.088 | 0.298 |
| Both | Cohort RR | Cohort | Rate Ratio | 2010 | 0.160 | 0.044 | 0.584 |
| Both | Cohort RR | Cohort | Rate Ratio | 2015 | 0.115 | 0.015 | 0.898 |
| Both | Cohort RR | Cohort | Rate Ratio | 2020 | 0.065 | 0.002 | 2.599 |
| Female | Net Drift | NA | NA | NA | -5.996 | -7.19 | -4.785 |
| Female | Local Drifts with Net Drift | Age | Percent per Year | <5 | -8.993 | -31.781 | 21.408 |
| Female | Local Drifts with Net Drift | Age | Percent per Year | 5~9 | -0.780 | -15.657 | 16.721 |
| Female | Local Drifts with Net Drift | Age | Percent per Year | 10~14 | -4.479 | -13.791 | 5.840 |
| Female | Local Drifts with Net Drift | Age | Percent per Year | 15~19 | -2.743 | -6.766 | 1.454 |
| Female | Local Drifts with Net Drift | Age | Percent per Year | 20~24 | -3.216 | -5.954 | -0.398 |
| Female | Local Drifts with Net Drift | Age | Percent per Year | 25~29 | -7.262 | -9.696 | -4.763 |
| Female | Local Drifts with Net Drift | Age | Percent per Year | 30~34 | -5.783 | -8.197 | -3.306 |
| Female | Local Drifts with Net Drift | Age | Percent per Year | 35~39 | -4.197 | -6.969 | -1.342 |
| Female | Local Drifts with Net Drift | Age | Percent per Year | 40~44 | -4.429 | -7.306 | -1.463 |
| Female | Local Drifts with Net Drift | Age | Percent per Year | 45~49 | -7.223 | -10.075 | -4.281 |
| Female | Local Drifts with Net Drift | Age | Percent per Year | 50~54 | -6.735 | -9.588 | -3.791 |
| Female | Local Drifts with Net Drift | Age | Percent per Year | 55~59 | -7.925 | -10.688 | -5.076 |
| Female | Local Drifts with Net Drift | Age | Percent per Year | 60~64 | -9.560 | -12.395 | -6.633 |
| Female | Local Drifts with Net Drift | Age | Percent per Year | 65~69 | -8.317 | -11.196 | -5.345 |
| Female | Local Drifts with Net Drift | Age | Percent per Year | 70~74 | -6.709 | -9.818 | -3.493 |
| Female | Local Drifts with Net Drift | Age | Percent per Year | 75~79 | -5.630 | -9.204 | -1.915 |
| Female | Local Drifts with Net Drift | Age | Percent per Year | 80~84 | -4.477 | -8.854 | 0.110 |
| Female | Local Drifts with Net Drift | Age | Percent per Year | ≥85 | -4.599 | -11.108 | 2.387 |
| Female | Longitudinal Age Curve | Age | Rate | <5 | 2.413 | 0.446 | 13.050 |
| Female | Longitudinal Age Curve | Age | Rate | 5~9 | 3.608 | 0.937 | 13.896 |
| Female | Longitudinal Age Curve | Age | Rate | 10~14 | 8.985 | 4.233 | 19.071 |
| Female | Longitudinal Age Curve | Age | Rate | 15~19 | 74.439 | 48.318 | 114.68 |
| Female | Longitudinal Age Curve | Age | Rate | 20~24 | 96.957 | 67.885 | 138.479 |
| Female | Longitudinal Age Curve | Age | Rate | 25~29 | 91.106 | 66.302 | 125.189 |
| Female | Longitudinal Age Curve | Age | Rate | 30~34 | 30.261 | 22.626 | 40.473 |
| Female | Longitudinal Age Curve | Age | Rate | 35~39 | 22.714 | 18.422 | 28.006 |
| Female | Longitudinal Age Curve | Age | Rate | 40~44 | 15.566 | 12.438 | 19.480 |
| Female | Longitudinal Age Curve | Age | Rate | 45~49 | 11.640 | 8.974 | 15.097 |
| Female | Longitudinal Age Curve | Age | Rate | 50~54 | 7.859 | 5.666 | 10.903 |
| Female | Longitudinal Age Curve | Age | Rate | 55~59 | 5.446 | 3.727 | 7.959 |
| Female | Longitudinal Age Curve | Age | Rate | 60~64 | 5.178 | 3.355 | 7.993 |
| Female | Longitudinal Age Curve | Age | Rate | 65~69 | 3.088 | 1.921 | 4.962 |
| Female | Longitudinal Age Curve | Age | Rate | 70~74 | 2.830 | 1.690 | 4.739 |
| Female | Longitudinal Age Curve | Age | Rate | 75~79 | 2.229 | 1.257 | 3.953 |
| Female | Longitudinal Age Curve | Age | Rate | 80~84 | 1.712 | 0.903 | 3.242 |
| Female | Longitudinal Age Curve | Age | Rate | ≥85 | 0.931 | 0.444 | 1.956 |
| Female | Period RR | Period | Rate Ratio | 2012 | 1.351 | 1.219 | 1.498 |
| Female | Period RR | Period | Rate Ratio | 2017 | 1 | 1 | 1 |
| Female | Period RR | Period | Rate Ratio | 2022 | 0.728 | 0.649 | 0.817 |
| Female | Cohort RR | Cohort | Rate Ratio | 1925 | 32.917 | 13.012 | 83.267 |
| Female | Cohort RR | Cohort | Rate Ratio | 1930 | 25.15 | 12.317 | 51.355 |
| Female | Cohort RR | Cohort | Rate Ratio | 1935 | 20.557 | 11.036 | 38.292 |
| Female | Cohort RR | Cohort | Rate Ratio | 1940 | 15.908 | 9.079 | 27.873 |
| Female | Cohort RR | Cohort | Rate Ratio | 1945 | 11.517 | 6.910 | 19.194 |
| Female | Cohort RR | Cohort | Rate Ratio | 1950 | 7.944 | 4.981 | 12.667 |
| Female | Cohort RR | Cohort | Rate Ratio | 1955 | 4.833 | 3.176 | 7.354 |
| Female | Cohort RR | Cohort | Rate Ratio | 1960 | 2.908 | 2.000 | 4.230 |
| Female | Cohort RR | Cohort | Rate Ratio | 1965 | 2.116 | 1.549 | 2.892 |
| Female | Cohort RR | Cohort | Rate Ratio | 1970 | 1.448 | 1.119 | 1.875 |
| Female | Cohort RR | Cohort | Rate Ratio | 1975 | 1 | 1 | 1 |
| Female | Cohort RR | Cohort | Rate Ratio | 1980 | 0.921 | 0.713 | 1.189 |
| Female | Cohort RR | Cohort | Rate Ratio | 1985 | 0.651 | 0.486 | 0.874 |
| Female | Cohort RR | Cohort | Rate Ratio | 1990 | 0.507 | 0.365 | 0.706 |
| Female | Cohort RR | Cohort | Rate Ratio | 1995 | 0.306 | 0.209 | 0.45 |
| Female | Cohort RR | Cohort | Rate Ratio | 2000 | 0.366 | 0.237 | 0.565 |
| Female | Cohort RR | Cohort | Rate Ratio | 2005 | 0.232 | 0.134 | 0.403 |
| Female | Cohort RR | Cohort | Rate Ratio | 2010 | 0.231 | 0.078 | 0.691 |
| Female | Cohort RR | Cohort | Rate Ratio | 2015 | 0.215 | 0.039 | 1.169 |
| Female | Cohort RR | Cohort | Rate Ratio | 2020 | 0.090 | 0.004 | 1.919 |
| Male | Net Drift | NA | NA | NA | -7.792 | -9.306 | -6.251 |
| Male | Local Drifts with Net Drift | Age | Percent per Year | <5 | -8.556 | -40.762 | 41.161 |
| Male | Local Drifts with Net Drift | Age | Percent per Year | 5~9 | -5.440 | -26.827 | 22.198 |
| Male | Local Drifts with Net Drift | Age | Percent per Year | 10~14 | -8.162 | -20.988 | 6.745 |
| Male | Local Drifts with Net Drift | Age | Percent per Year | 15~19 | -6.397 | -11.243 | -1.285 |
| Male | Local Drifts with Net Drift | Age | Percent per Year | 20~24 | -3.075 | -6.356 | -0.321 |
| Male | Local Drifts with Net Drift | Age | Percent per Year | 25~29 | -6.260 | -9.390 | -3.022 |
| Male | Local Drifts with Net Drift | Age | Percent per Year | 30~34 | -5.707 | -8.994 | -2.300 |
| Male | Local Drifts with Net Drift | Age | Percent per Year | 35~39 | -6.933 | -10.406 | -3.326 |
| Male | Local Drifts with Net Drift | Age | Percent per Year | 40~44 | -7.179 | -10.601 | -3.626 |
| Male | Local Drifts with Net Drift | Age | Percent per Year | 45~49 | -8.473 | -11.56 | -5.279 |
| Male | Local Drifts with Net Drift | Age | Percent per Year | 50~54 | -8.234 | -10.957 | -5.428 |
| Male | Local Drifts with Net Drift | Age | Percent per Year | 55~59 | -10.580 | -13.060 | -8.028 |
| Male | Local Drifts with Net Drift | Age | Percent per Year | 60~64 | -11.748 | -14.166 | -9.261 |
| Male | Local Drifts with Net Drift | Age | Percent per Year | 65~69 | -9.703 | -12.072 | -7.269 |
| Male | Local Drifts with Net Drift | Age | Percent per Year | 70~74 | -9.447 | -11.979 | -6.843 |
| Male | Local Drifts with Net Drift | Age | Percent per Year | 75~79 | -8.053 | -11.134 | -4.865 |
| Male | Local Drifts with Net Drift | Age | Percent per Year | 80~84 | -7.721 | -11.83 | -3.421 |
| Male | Local Drifts with Net Drift | Age | Percent per Year | ≥85 | -5.615 | -13.489 | 2.975 |
| Male | Longitudinal Age Curve | Age | Rate | <5 | 4.970 | 0.342 | 72.122 |
| Male | Longitudinal Age Curve | Age | Rate | 5~9 | 8.659 | 1.621 | 46.271 |
| Male | Longitudinal Age Curve | Age | Rate | 10~14 | 12.396 | 4.274 | 35.951 |
| Male | Longitudinal Age Curve | Age | Rate | 15~19 | 183.562 | 106.861 | 315.317 |
| Male | Longitudinal Age Curve | Age | Rate | 20~24 | 221.304 | 139.316 | 351.541 |
| Male | Longitudinal Age Curve | Age | Rate | 25~29 | 185.469 | 122.471 | 280.870 |
| Male | Longitudinal Age Curve | Age | Rate | 30~34 | 60.001 | 41.308 | 87.154 |
| Male | Longitudinal Age Curve | Age | Rate | 35~39 | 45.780 | 35.652 | 58.785 |
| Male | Longitudinal Age Curve | Age | Rate | 40~44 | 32.952 | 25.538 | 42.517 |
| Male | Longitudinal Age Curve | Age | Rate | 45~49 | 27.652 | 20.481 | 37.334 |
| Male | Longitudinal Age Curve | Age | Rate | 50~54 | 20.965 | 14.725 | 29.850 |
| Male | Longitudinal Age Curve | Age | Rate | 55~59 | 15.088 | 10.121 | 22.494 |
| Male | Longitudinal Age Curve | Age | Rate | 60~64 | 14.066 | 9.074 | 21.803 |
| Male | Longitudinal Age Curve | Age | Rate | 65~69 | 8.193 | 5.138 | 13.064 |
| Male | Longitudinal Age Curve | Age | Rate | 70~74 | 6.659 | 4.035 | 10.991 |
| Male | Longitudinal Age Curve | Age | Rate | 75~79 | 4.495 | 2.607 | 7.749 |
| Male | Longitudinal Age Curve | Age | Rate | 80~84 | 2.999 | 1.618 | 5.560 |
| Male | Longitudinal Age Curve | Age | Rate | ≥85 | 1.278 | 0.595 | 2.747 |
| Male | Period RR | Period | Rate Ratio | 2012 | 1.424 | 1.269 | 1.599 |
| Male | Period RR | Period | Rate Ratio | 2017 | 1 | 1 | 1 |
| Male | Period RR | Period | Rate Ratio | 2022 | 0.633 | 0.554 | 0.722 |
| Male | Cohort RR | Cohort | Rate Ratio | 1925 | 84.92 | 29.952 | 240.768 |
| Male | Cohort RR | Cohort | Rate Ratio | 1930 | 74.868 | 37.626 | 148.972 |
| Male | Cohort RR | Cohort | Rate Ratio | 1935 | 47.647 | 26.450 | 85.831 |
| Male | Cohort RR | Cohort | Rate Ratio | 1940 | 33.522 | 19.720 | 56.982 |
| Male | Cohort RR | Cohort | Rate Ratio | 1945 | 20.578 | 12.567 | 33.696 |
| Male | Cohort RR | Cohort | Rate Ratio | 1950 | 12.426 | 7.842 | 19.690 |
| Male | Cohort RR | Cohort | Rate Ratio | 1955 | 7.416 | 4.843 | 11.355 |
| Male | Cohort RR | Cohort | Rate Ratio | 1960 | 3.561 | 2.405 | 5.274 |
| Male | Cohort RR | Cohort | Rate Ratio | 1965 | 2.424 | 1.720 | 3.416 |
| Male | Cohort RR | Cohort | Rate Ratio | 1970 | 1.508 | 1.122 | 2.027 |
| Male | Cohort RR | Cohort | Rate Ratio | 1975 | 1 | 1 | 1 |
| Male | Cohort RR | Cohort | Rate Ratio | 1980 | 0.716 | 0.521 | 0.983 |
| Male | Cohort RR | Cohort | Rate Ratio | 1985 | 0.487 | 0.333 | 0.713 |
| Male | Cohort RR | Cohort | Rate Ratio | 1990 | 0.398 | 0.258 | 0.613 |
| Male | Cohort RR | Cohort | Rate Ratio | 1995 | 0.255 | 0.156 | 0.418 |
| Male | Cohort RR | Cohort | Rate Ratio | 2000 | 0.291 | 0.168 | 0.503 |
| Male | Cohort RR | Cohort | Rate Ratio | 2005 | 0.132 | 0.065 | 0.267 |
| Male | Cohort RR | Cohort | Rate Ratio | 2010 | 0.124 | 0.025 | 0.605 |
| Male | Cohort RR | Cohort | Rate Ratio | 2015 | 0.075 | 0.005 | 1.056 |
| Male | Cohort RR | Cohort | Rate Ratio | 2020 | 0.051 | 0.001 | 4.858 |

**Table S2 JoinPoint regression results**

| **Cohort** | **Range** | **Segment** | **Lower Endpoint** | **Upper Endpoint** | **APC/AAPC** | **Lower CI** | **Upper CI** | **Test Statistic** | ***P*-Value** |
| --- | --- | --- | --- | --- | --- | --- | --- | --- | --- |
| both - 0 Joinpoints | Full Range | 1 | 2010 | 2024 | -7.661 | -8.808 | -6.501 | -13.784 | < 0.001 |
| male - 0 Joinpoints | Full Range | 1 | 2010 | 2024 | -8.242 | -9.504 | -6.961 | -13.411 | < 0.001 |
| female - 0 Joinpoints | Full Range | 1 | 2010 | 2024 | -6.029 | -6.974 | -5.075 | -13.296 | < 0.001 |

**Table S3 FleXScan results**

| **Clusters type** | **Gathering area** | **Observed cases** | **Expected cases** | **RR** | **LLR** | ***P*** |
| --- | --- | --- | --- | --- | --- | --- |
| I | Jiaxiang | 5197 | 3941 | 1.32 | 201.82 | ＜0.001 |
| II | Weishan | 4314 | 3395 | 1.27 | 124.95 | ＜0.001 |
| II | Qufu、Shishui | 7392 | 6268 | 1.18 | 112.22 | ＜0.001 |
| II | Wenshang | 3 977 | 3706 | 1.07 | 10.54 | ＜0.001 |

**Figure S1 Local Drifts with confidence interval removed**

**
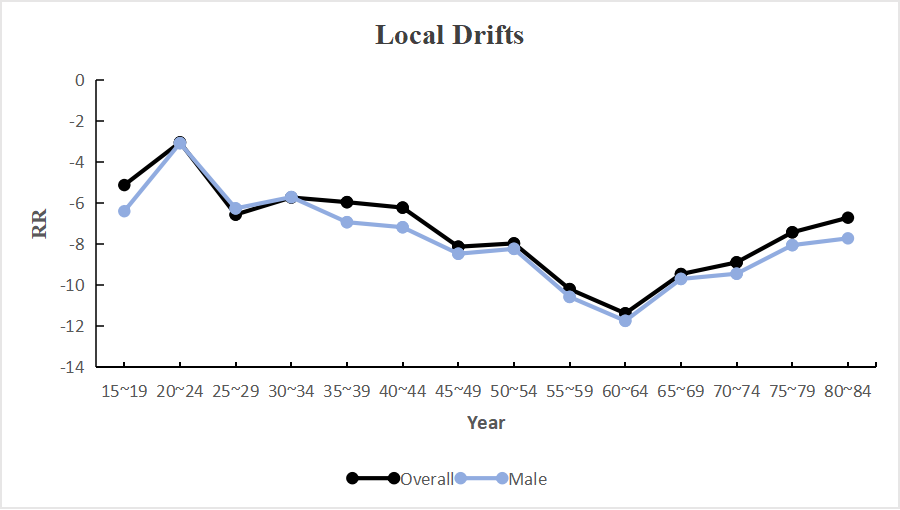
**
